# The concentration of maternal sacubitril/valsartan transferred into human milk is negligible

**DOI:** 10.3389/fpubh.2024.1389513

**Published:** 2024-05-22

**Authors:** Sirin Falconi, Abiodun Okimi, Shaun Wesley, Pooja Sethi, Palika Datta, Kaytlin Krutsch

**Affiliations:** ^1^School of Medicine, Texas Tech University Health Sciences Center, Amarillo, TX, United States; ^2^Department of Obstetrics and Gynecology, School of Medicine and Dentistry, University of Rochester Medical Center, Rochester, NY, United States; ^3^Department of Cardiology, School of Medicine, Texas Tech University Health Sciences Center, Lubbock, TX, United States; ^4^Department of Obstetrics and Gynecology, School of Medicine, Texas Tech University Health Sciences Center, Amarillo, TX, United States

**Keywords:** heart failure, Entresto, lactation, pharmacology, peripartum cardiomyopathy (PPCM), maternal health

## Abstract

**Background:**

Peripartum cardiomyopathy (PPCM) is a common cause of heart failure (HF) in the peripartum. Some medications are considered safe while breastfeeding. However, sacubitril/valsartan (Entresto), while efficacious, is not recommended in breastfeeding women due to concerns about adverse infant development, and no published data suggest otherwise.

**Objectives:**

This study aimed to assess the transfer of sacubitril/valsartan into human milk and evaluate the infant’s risk of drug exposure.

**Methods:**

The InfantRisk Human Milk Biorepository released samples and corresponding health information from five breastfeeding maternal–infant dyads exposed to sacubitril/valsartan. Sacubitril, valsartan, and LBQ657 (sacubitril active metabolite) concentrations were determined using liquid chromatography-mass spectrometry (LC/MS/MS) from timed samples 0, 1, 2, 4, 6, 8, 10, and 12 h following medication administration at steady state conditions.

**Results:**

Valsartan levels were below the detection limit of 0.19 ng/mL in all milk samples. Sacubitril was measurable in all milk samples of the five participants, peaking 1 h after drug administration at a mean concentration of 1.52 ng/mL for a total infant dose of 0.00049 mg/kg/12 h and a relative infant dose (RID) calculated at 0.01%. The maximum concentration of its active metabolite LBQ657 in the milk samples was observed 4 h after medication administration and declined over the remaining 12-h dosing interval, for an average concentration of 9.5 ng/mL. The total infant dose was 0.00071 mg/kg/12 h, and the RID was 0.22%. Two mothers reported continuing to breastfeed while taking sacubitril/valsartan; both mothers stated observing no negative effects in their breastfed infants.

**Conclusion:**

The transfer of sacubitril/valsartan into human milk is minimal. These concentrations are unlikely to pose a significant risk to breastfeeding infants, with a combined calculated RID of <0.25%, which is far lower than the industry safety standards (RID <10%).

## Introduction

Human milk is the ideal source of nutrition for infants; breastfeeding is currently encouraged in mothers for the first 2 years postpartum (1). The 2022 American Academy of Pediatrics’ extension of lactation recommendations from 12 to 24 months is rooted in the dose-intensive amplification of maternal health outcomes rather than infant benefits (1). Several studies discuss the protective association between breastfeeding and short- and long-term maternal cardiovascular risk factors such as hypertension, stroke, type 2 diabetes, obesity, and metabolic syndromes, culminating in decreased cardiovascular disease incidence and mortality (2, 3). These benefits may be explained by the maternal reset hypothesis, where lactation leads to the reestablishment of glucose and lipid homeostasis after pregnancy (3, 4). Increased lifetime lactation is associated with reduced primary and secondary cardiovascular disease prevalence (2).

Heart failure (HF) is a complex syndrome resulting from any structural or functional impairment to ventricular filling or ejection of blood from the heart (5). Common causes include ischemic heart disease, hypertension, valvular disease, cardiomyopathies, and peripartum cardiomyopathy (PPCM) (5). PPCM is an idiopathic dilated cardiomyopathy that presents as a new onset of left ventricular dysfunction occurring toward the end of pregnancy or following delivery without any known cause (6, 7). Risk factors include multiple pregnancies, ethnicity, smoking, diabetes, hypertension, pre-eclampsia, the age of the mother, prolonged use of beta-agonists, and viral infections (7). The incidence of PPCM doubled between 1990 and 2000 and increased another 38% from 2004 to 2011 (8). Cardiovascular disease now accounts for approximately 30% of maternal mortality in the United States. Cardiomyopathy accounts for 50 to 65% of these cases, underscoring the major impact of PPCM and the potential impact of medication treatments on maternal mortality (9, 10).

Therapeutic interventions for maternal HF and PPCM aim to modify risk factors, treat structural changes, and reduce symptoms, morbidity, and mortality. Sacubitril was the first Food and Drug Administration (FDA)-approved agent of the angiotensin receptor neprilysin inhibitor (ARNI) drug class in combination with valsartan, an angiotensin receptor blocker (ARB) (5, 11). Sacubitril inhibits neprilysin, an enzyme responsible for degrading vasoactive peptides (e.g., natriuretic peptide, adrenomedullin, and bradykinin), prolonging vasodilation, diuresis, natriuresis, and inhibiting fibrosis. Valsartan, an ARB, is used in conjunction with sacubitril to inhibit the effect of excess angiotensin II (ANG II) (5, 11). The drug combination simultaneously counteracts ANG II’s negative effects while retaining the advantages of increased NPs (5, 11). Sacubitril/valsartan decreases the risk of hospitalization and cardiovascular death in patients with chronic HF with reduced ejection fraction with the New York Heart Association (NYHA) classes II, III, or IV versus valsartan alone, as suggested by the Prospective Comparison of ARNI with ARB Global Outcomes in HF with Preserved Ejection Fraction (PARAGON-HF) and the Prospective Comparison of ARNI with ARB on the Management of Heart Failure with Preserved Ejection Fraction (PARAMOUNT-HF) (6, 11).

Medications are considered “on-label” for breastfeeding women when used for an FDA-approved indication independent of lactation status (12). Although sacubitril/valsartan is FDA-approved and known to be beneficial to treat HF, and breastfeeding is known to reduce adverse cardiovascular outcomes on a population scale, the drug is not recommended by LactMed, the FDA, the American College of Cardiology (ACC), or the American Heart Association (AHA) in breastfeeding women. This recommendation is due to concerns about unknown infant adverse events, such as hypotension, and the lack of published data, including the concentrations of sacubitril/valsartan transferred in milk (6, 7, 13). Though these guidelines support using angiotensin-converting enzyme inhibitors while breastfeeding, ARBs are not mentioned (14). Research studies have yet to be conducted on the maternal use of sacubitril or valsartan and their transfer into breast milk or on the effects on breastfed infants.

It is plausible that postpartum women suffering from primary cardiovascular diseases such as HF could benefit from the pleiotropic effects of lactation. However, many women requiring chronic medication treatment are advised to discontinue breastfeeding out of an abundance of caution due to potential infant drug exposure and resulting adverse effects (15). This study aims to quantify the amount of drug present in the milk of mothers taking the HF medication, sacubitril/valsartan, and report any observed effects on their breastfed infants.

## Materials and methods

Sacubitril/valsartan is available as a fixed-dose combination, Entresto, taken twice daily. The InfantRisk Human Milk Biorepository (HMB) was searched for participants taking sacubitril/valsartan oral tablets at the time of milk donation. Each participant provided written (electronic) informed consent for research participation and publication through the HMB (Texas Tech University Health Sciences Center Amarillo IRB no. A21-4214). The HMB collects and aggregates milk samples from mothers observationally taking medications of research interest. All mothers were advised upon contact with the InfantRisk Center to cease breastfeeding prior to sacubitril/valsartan initiation. The deidentified materials, including milk samples and self-reported medical questionnaires for five participants, matched this query and were released from the biorepository. All participants were instructed to provide milk samples under steady-state conditions at 0, 1, 2, 4, 6, 8, 10, and 12 h after administering their medications. Mothers were advised to express milk from both breasts, gently mix, and freeze in a collection tube prior to overnight shipment to our facility. Upon receipt, the milk samples were stored at −80°C until further analysis.

Sacubitril is a prodrug converted by esterases in the liver to the active metabolite LBQ657. Sacubitril, LBQ657, and valsartan were measured using an Agilent Ultivo mass spectrometer (MS/MS). A Phenomenex Luna C18 column was used for the separation phase, and chromatographic separation was performed using gradient elution, with mobile phase A as 0.1% formic acid in water and mobile phase B as 0.1% formic acid in acetonitrile, which was delivered at a continuous flow rate of 0.4 mL/min. Electrospray ionization with multiple reaction monitoring in positive mode was used. For sacubitril, valsartan and LBQ657 and internal standard (IS) sacubutril-d_4_ and valsartan-d_9_ at the m/z transitions 412.2–266.1, 436.2–291, 384.1–266.1 and for IS 416.2–266 and 445.3–207.1 were selected. The calibration curve was made using blank human milk in the range of 0.09–50 ng/mL for all the analytes. The limit of detection for sacubitril and its metabolite LBQ657 was 0.09 ng/mL, whereas for valsartan it was 0.19 ng/mL. The quality control samples (low, medium, and high concentrations) were evaluated against the calibration curve. All samples were prepared using the deproteination process. The linearity of the calibration curve was evaluated by the coefficient of determination (r^2^) of the linear regression analysis of the concentration-response data using a weight of 1/x^2^, where x is the concentration. The mean correlation coefficient of the weighted calibration curves generated during the validation was ≥0.99. The analytical method was validated with respect to specificity, linearity, sensitivity, matrix effects, and recovery. Additional information can be found in the Supplementary materials.

## Results

**Figure 1:**
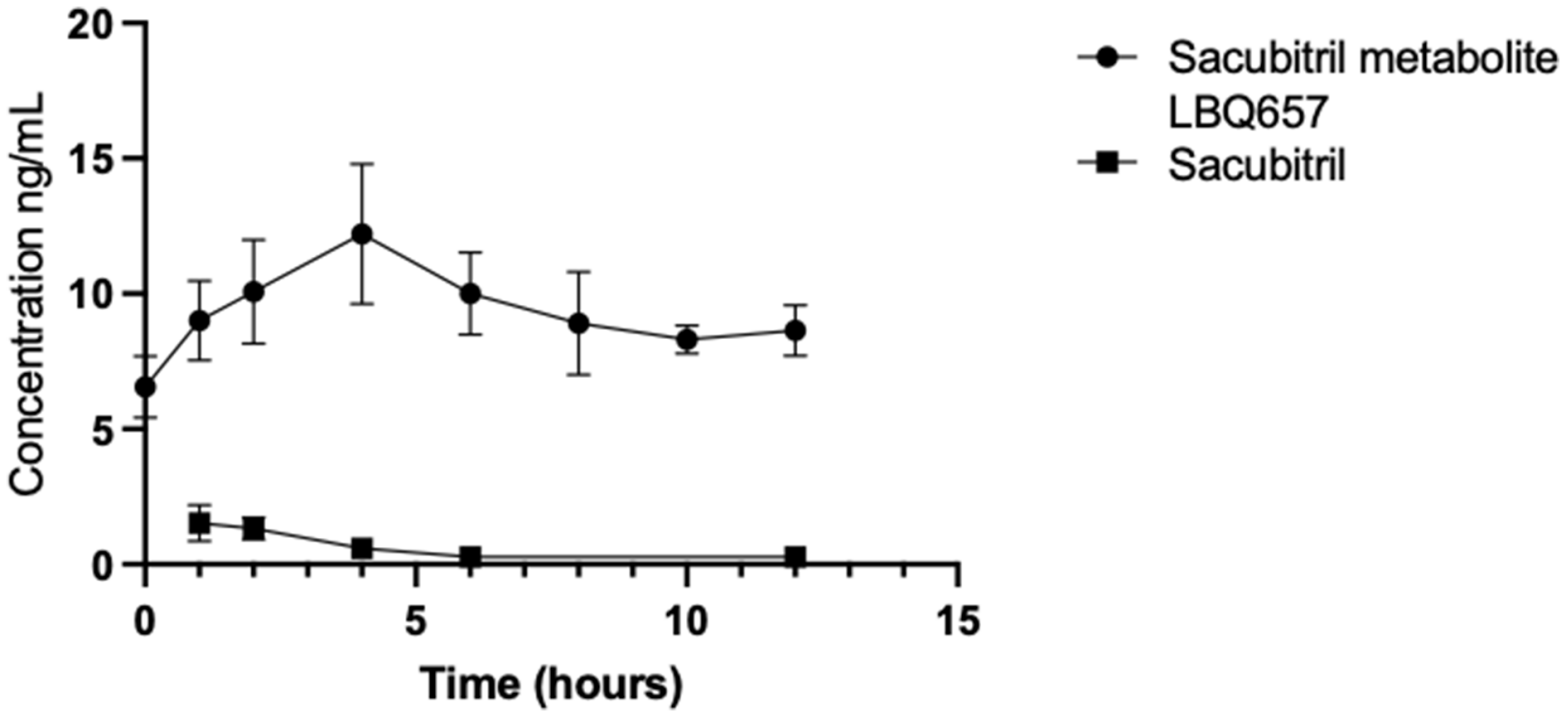
Drug concentrations in timed human milk samples (*n* = 5) were quantified using LC/MS/MS. The 12-h time-concentration profile characterizes the maternal transfer of drug residues into milk for sacubitril and its active metabolite, LBQ657. Valsartan was not quantified in any milk samples at a detection level of 0.19 ng/mL.

This study investigated the concentrations of sacubitril, LBQ657, and valsartan in milk samples obtained from five biorepository participants. These individuals were previously prescribed sacubitril/valsartan 24 mg/26 mg orally twice daily at the time of milk collection. All participants initiated sacubitril/valsartan recently in the peripartum period. Valsartan levels were found to be below the detection limit of 0.19 ng/mL in all milk samples. Figure 1 demonstrates the 12-h concentration-time profile of sacubitril and LBQ657; Table 1 presents the pharmacokinetic analysis and calculated infant exposures. Sacubitril, the prodrug, was measurable in all five participants (16 of 40 samples), peaking 1 h after drug administration at a mean concentration of 1.52 ng/mL (range: 0.42–2.68 ng/mL), total infant dose of 0.00049 mg/kg/12 h, and RID of 0.01%. The maximum concentration of its active metabolite, LBQ657 was observed 4 h after medication administration and declined over the remaining 12-h dosing period (Figure 1). The total infant dose was 0.00071 mg/kg/12 h assuming a milk consumption of 150 mL/kg/day using the average concentration (16).

**Table 1:** Pharmacokinetic parameters of sacubitril, its active metabolite LBQ657, and valsartan.

Parameters (units) (*n* = 5)	Value (sacubitril)	Value (LBQ657)	Valsartan
**Calculated milk parameters**			
AUC (ng.hr./mL)	5.96	114.9	ND
C_avg_ (ng/mL)	0.4966	9.5	ND
C_max_ (ng/mL)	1.52	12.2	ND
T_max_ (h)	1	4	ND
Infant dose (mg/kg/12 h)	0.00049	0.00071	–
RID (%)	0.01	0.22	–
**Known drug properties**			
Molecular weight (g/mol)	411.5	383.4	435.5
Protein binding	>94%	>94%	>94%
Volume of distribution (L)	75	–	103
Half-life (h, adult)	1.4	11.5	9.9
Oral bioavailability	>60%	–	>23%*
Partition coefficient (CLogP)	4.47	–	4.86

AUC: Area under curve, C_avg_: average drug concentration, C_max_: maximum drug concentration, RID: relative infant dose, ND: not detected.*The oral bioavailability of valsartan in sacubitril/valsartan is higher than in valsartan-only products. The area under the curve was calculated using the log-linear trapezoidal method for 0–24 h and the average concentration [relative infant dose was calculated using the formula: relative infant dose (%) = estimated daily infant dose via breast milk assuming a milk intake of 150 mL/kg/day (mg/kg/day)/maternal dose (mg/kg/day) × 100].

The relative infant dose (RID) of LBQ657 was calculated using various approaches to simulate multiple situations. Using the average concentration and peak concentration, %RID was 0.22 and 0.27, respectively, with a denominator of maternal dose per kilogram. To simulate the “worst-case scenario,” we used the highest observed LBQ657 concentration level (19.6 ng/mL) to calculate a RID of 0.46%.

The results from the participant health questionnaire are summarized in Table 2. Two participants chose not to withhold breastmilk from their infants after initiating sacubitril/valsartan. The two infants were subsequently exposed to sacubitril/valsartan via milk, one via a full diet of breastmilk and the other partially breastfed. During the period of less than 2 weeks from drug initiation to HMB reporting, neither mother observed any negative adverse effects in their child. One mother reported that her child exceeded expectations in developmental milestones such as height and weight, and the other reported surpassing all others except for verbal communication. One mother reported that she had been prescribed sacubitril/valsartan within the first month postpartum but delayed initiating the medication to the detriment of her own health until her child was 6 months old in order to continue breastfeeding.

**Table 2:** Mother–infant demographics.

Patient	Maternal age (years)	Race	Medical history	Other medications	Child’s age range	Participant’s body weight (lbs)
1	39	Hispanic	Blood clotting disorder, anemia, breast augmentation, underproduction of breastmilk	Rivaroxaban, furosemide, enoxaparin, captopril, amlodipine, cephalexin, acetaminophen	11 days-4 weeks	119
2	26	Caucasian	Anxiety, depression, underproduction of breastmilk	Carvedilol, spironolactone, escitalopram	4–6 months	160
3	24	Caucasian	Anxiety, arrythmia, latent tuberculosis	Metoprolol, spironolactone	4–6 months	176
4	34	Caucasian	Cancer, gestational diabetes, hypertension, anxiety, migraines	Empagliflozin, spironolactone, metoprolol, buspirone, oxybutynin, bupropion, pantoprazole, torsemide	12–23 months	225
5	35	Caucasian	Postpartum anxiety, postpartum depression, gestational diabetes, gestational hypothyroidism	Metoprolol	4–6 months	145

Data extracted from the InfantRisk Human Milk Biorepository maternal–infant health questionnaires, detailing key parameters as reported by the participant such as the unique patient identifier, maternal age at the time of childbirth, racial background, previous medical history, concurrent medications at the time of milk sample expression, and the age range of the child.

## Discussion

There are no previous studies evaluating the transfer of any NEP inhibitor (e.g., sacubitril) into human milk. A single case series has reported the limited transfer of one ARB, candesartan, into human milk (25). This scarcity of data restricts the use of these drug classes in breastfeeding mothers suffering from HF. The percent RID represents the percentage of the weight-adjusted dose of sacubitril/valsartan that an infant would be exposed to via a complete diet of breast milk. The RID calculations suggest minimal exposure to sacubitril and its active metabolite, LBQ657, through breastfeeding, even under worst-case assumptions. This study reports a 0.01 and 0.22% RID for sacubitril and LBQ657, respectively, and undetectable valsartan found in human milk. These results demonstrate that infant exposure to sacubitril and valsartan drug residues via breastmilk is negligible, well below the standard 10% RID threshold for safety (16). With minimal transfer into breastmilk, sacubitril and valsartan may be suggested as viable treatment option(s) for breastfeeding mothers.

The reason for low levels of drug transfer into human milk is likely associated with the pharmacokinetics of the drug. Upon oral administration, sacubitril and valsartan are rapidly absorbed and reach maximum plasma concentration (Cmax) with a median time of 0.5 and 1.5 h, respectively (17). Sacubitril is quickly esterified to LBQ657, with a median time to peak drug concentration (Tmax) of 2 h for the metabolite. Our findings in milk parallel these known serum pharmacokinetic parameters. LBQ657 is not metabolized further to a significant extent. Valsartan is minimally metabolized (18, 19). The formulation of valsartan available in Entresto is more bioavailable than other preparations. The valsartan in Entresto 24 mg/26 mg tablets is equivalent to other marketed tablet formulations of valsartan 40 mg (18, 19). Once in the plasma, sacubitril, LBQ657, and valsartan are extensively protein-bound (96–97%), contributing to a low-free fraction of the drug available for transfer into breast milk. Plasma concentrations of sacubitril, LBQ657, and valsartan are metabolized with a mean elimination half-life of 1.4 h, 11.5 h, and 9.9 h, respectively (17, 18). In accordance with previously observed plasma concentrations, our data suggest a corresponding rapid decline in milk levels over a period of 12 h as well, further supporting parallel but muted drug concentrations in human milk.

Caution must be taken when relying on RIDs alone to guide risk management in breastfeeding infants of mothers taking medications. Although RID is a useful parameter, it only estimates exposure to the infant and does not account for individualized infant organogenesis, metabolic capacity, or fragility. Although rare, the most common adverse effect attributed to sacubitril is angioedema, while the most common adverse effects found with valsartan use include hypotension, hyperkalemia, cough, and worsening renal function (11). There is no previous information regarding adverse effects in lactating infants of mothers taking sacubitril/valsartan. Pregnant and lactating women were excluded from phase I through III trials (5, 18). In our study, two mothers continued breastfeeding while on valsartan/sacubitril and did not report any observed symptoms in their breastfed infants. The HMB does not collect longitudinal infant outcomes; therefore, long-term infant safety remains unknown. The issue of maternal medication adherence during lactation has often been overlooked, despite documented instances of non-compliance with antibiotics and antidepressants. This oversight highlights the need for greater attention to medication adherence among lactating mothers to ensure optimal treatment outcomes for maternal–infant health (20, 21). The decision of one of our participants to knowingly delay vital medications due to the belief that sacubitril/valsartan and breastfeeding were mutually exclusive behaviors demonstrates an unfortunate reciprocal connection between maternal medications and breastfeeding.

Breastfeeding in women with HF has historically been a controversial topic. The European Society of Cardiology PPCM guidelines, as recent as 2018, discourage lactation in patients with severe HF due to the high metabolic demand and to enable optimal medication treatment (though most medications are compatible with breastfeeding) (22). In 2019, the position was modified, concluding that many women with PPCM tolerate breastfeeding and that individualized risk–benefit discussions regarding breastfeeding are necessary in every case, including the many physical and psychological benefits conferred to the breastfeeding dyad (6, 7). Though it was previously postulated that vasoinhibin, an antiangiogenic metabolite of prolactin, is associated with oxidative stress resulting in endothelial damage, multiple studies have failed to confirm this hypothesis (7, 23, 24). Though there is much left to learn about the relationship between lactation and cardiovascular health, the paucity of realized data associated with increased cardiovascular risk with lactation must be weighed against the overwhelming benefit breastfeeding has for the maternal–infant dyad. However, to effectively consider the risks and benefits of medication use while breastfeeding, the amount of drug transferred into human milk must be evaluated for each drug.

This study is limited by small sample size, a lack of corresponding maternal plasma samples, and drug exposures limited to the sacubitril/valsartan 24 mg/26 mg starting dose, much lower than the target maintenance dose of sacubitril 97 mg/valsartan 103 mg twice daily. It is important to acknowledge that the small sample size of five participants may limit the generalizability of our findings. Potential inter-individual variability in drug transfer and metabolism underscores the need for further investigations to validate and expand upon our results. However, our report is the first to produce information regarding sacubitril/valsartan use and lactation. In a field where clinical trials often fail due to lack of enrollment or are limited to case reports, this study evaluated five women taking sacubitril/valsartan collected over a 12-month period. Our study begins an evidence-informed conversation regarding the safety of sacubitril/valsartan in breastfeeding mothers with HF and their infants. By offering reassurance of safety for the breastfed infant, the previously competing interests of maternal medication treatment and breastfeeding can be considered independently.

## Conclusion

The results of this study suggest that the transfer of sacubitril and valsartan is minimal and unlikely to pose a significant risk to breastfeeding infants using established standards. In five lactating women who took sacubitril/valsartan, two infants were exposed to the drug residues via milk without any observed negative adverse effects. The undetectable valsartan, 0.01% RID for sacubitril, and 0.22% RID for sacubitril’s active metabolite (LBQ657) are comparable to or lower than current infant drug exposures for first-line treatments for HF in breastfeeding mothers, suggesting sacubitril and/or valsartan are feasible treatment options for breastfeeding mothers. An infant’s risk of minimal drug residue exposure must be weighed against the benefits of breastfeeding to both mother and child when determining the best course of action in treating HF in the lactating mother.

## Data availability statement

The raw data supporting the conclusions of this article will be made available by the authors, without undue reservation.

## Ethics statement

The studies involving humans were approved by Texas Tech University Health Sciences Center Amarillo IRB. The studies were conducted in accordance with the local legislation and institutional requirements. The human samples used in this study were acquired from materials were released from the InfantRisk Human Milk Biorepository. Written informed consent for participation was not required from the participants or the participants’ legal guardians/next of kin in accordance with the national legislation and institutional requirements.

## Author contributions

SF: Writing – original draft, Writing – review & editing. AO: Writing – original draft, Writing – review & editing. SW: Writing – original draft, Writing – review & editing. PS: Writing – original draft, Writing – review & editing. PD: Data curation, Formal analysis, Investigation, Methodology, Software, Validation, Visualization, Writing – original draft, Writing – review & editing. KK: Conceptualization, Formal analysis, Funding acquisition, Investigation, Methodology, Project administration, Supervision, Writing – original draft, Writing – review & editing.

## Glossary

ACC: American College of Cardiology
AHA: American Heart Association
ANG II: Angiotensin II
ARB: Angiotensin receptor blocker
ARNI: Angiotensin receptor neprilysin inhibitor
Cmax: Maximum plasma concentration
FDA: Food and Drug Administration
HF: Heart failure
HMB: Human milk biorepository
HTN: Hypertension
IRB: Institutional Review Board
IS: Internal standard
MS/MS: Agilent Ultivo mass spectrometer
NEP: Neprilysin
NP: Natriuretic peptide
NYHA: New York Heart Association
PARAGON-HF: Prospective comparison of ARNI with ARB global outcomes in HF with preserved ejection fraction
PARAMOUNT-HF: Prospective comparison of ARNI with ARB on management of heart failure with preserved ejection fraction
PPCM: Peripartum cardiomyopathy
r^2^: Coefficient of determination
RID: Relative infant dose
Tmax: Time to peak drug concentration
