# Corrigendum: The concentration of maternal sacubitril/valsartan transferred into human milk is negligible

**DOI:** 10.3389/fpubh.2024.1517339

**Published:** 2024-11-13

**Authors:** Sirin Falconi, Abiodun Okimi, Shaun Wesley, Pooja Sethi, Palika Datta, Kaytlin Krutsch

**Affiliations:** ^1^School of Medicine, Texas Tech University Health Sciences Center, Amarillo, TX, United States; ^2^Department of Obstetrics and Gynecology, School of Medicine and Dentistry, University of Rochester Medical Center, Rochester, NY, United States; ^3^Department of Cardiology, School of Medicine, Texas Tech University Health Sciences Center, Lubbock, TX, United States; ^4^Department of Obstetrics and Gynecology, School of Medicine, Texas Tech University Health Sciences Center, Amarillo, TX, United States

**Keywords:** heart failure, entresto, lactation, pharmacology, peripartum cardiomyopathy (PPCM), maternal health

In the published article, there was an error. The currently published article does not acknowledge a previously published study. We would like to add the missing data and properly reference the earlier work.

A correction has been made to **Discussion**, paragraph one.

This sentence previously stated:

“There are no previous studies evaluating the transfer of any NEP inhibitor (e.g., sacubitril) or ARB (e.g., valsartan) into human milk, limiting the use of these drug classes in breastfeeding mothers suffering from HF.”

The corrected sentence appears below:

“There are no previous studies evaluating the transfer of any NEP inhibitor (e.g., sacubitril) into human milk. A single case series has reported the limited transfer of one ARB, candesartan, into human milk (25). This scarcity of data restricts the use of these drug classes in breastfeeding mothers suffering from HF.”

The authors apologize for this error and state that this does not change the scientific conclusions of the article in any way. The original article has been updated.
