# Supplementary material for: The concentration of maternal sacubitril/valsartan transferred into human milk is negligible
Source: Front Public Health. 2024 May 22;12:1389513. doi: 10.3389/fpubh.2024.1389513 (PMC11150817; doi:10.3389/fpubh.2024.1389513)
Supplement: Supplementary file 1 [file Data_Sheet_1.PDF]

Additional information:

The developed biological analysis method has been thoroughly validated including the evaluation of selectivity, linearity, precision and accuracy, recovery rate and matrix effect.

Selectivity:

Blank matrix samples from lactating women (not taking any medications and containing no analyte) were run to obtain the selectivity of the method. To ensure that there is no interference between analyte transitions, neat samples of single analyte without the matrix were run. No significant interference was observed in the respective MRMs of each analyte.

Linearity, accuracy and precision:

For calibration curves, a weighed ($1/x^2$) least squares linear regression method was used to construct standard calibration curves ranging from 0.19 to 50 ng/mL, with correlation coefficients equal to 0.99 for all batches analyzed represented in Table S1. Quality control levels samples (LQC, MQC, HQC) were run with each calibration batch. Inter and intra-day runs were performed to determine the accuracy and precision of the method. The accuracy of the three analytes ranged from 85 – 115% and the precision values ranged from 1.79 – 6.45 %. Table 2 shows the accuracy and precision of the samples run at different concentrations.

Recovery and matrix effects:

The recovery of three analytes was performed by comparing the analytical results of extracted samples with corresponding extracts of blank milk spiked with analyte post-extraction (which represents 100% recovery). Matrix effects were validated using blank milk from 5 different sources and was evaluated by adding the pure analyte in water and processed in the same way as the milk samples prepared for different concentrations. Recoveries of all analytes were relatively high more than 85% using the milk matrix and due to high recovery, there was minimal matrix effect observed.

Table S1:

Calibration curve calculations

**Analyte**	**Regression equation**	**Correlation coefficient**	**Range (ng/mL)**

Valsartan	Y= 1.436X + 0.0119	0.999	0.19 - 50
Sacubitril	Y= 0.422X + 0.0050	0.999	0.09 - 50
LBQ657	Y= 0.153X + 0.0059	0.998	0.09 - 50

Table S2: Inter-run and Intra-run accuracy and precision values of the lowest, middle, and highest concentrations of triple analytes in standard curve. The accuracy was calculated as a percentage of measured concentration over nominal concentration. Precision was calculated as a percentage of relative standard deviations (RSD).

**Analyte**	**Added Conc. (ng/ml)**	**Interday run**				**Intraday run**		
		**Measured Conc. (ng/ml)**	**Accuracy (%)**	**Precision (RSD %)**		**Measured Conc. (ng/ml)**	**Accuracy (%)**	**Precision (RSD %)**
**Valsartan**	0.39	0.43	110	6.45		0.45	115	5.7
	3.12	3.28	105	3.17		3.45	110.5	4.23
	25	25.13	100.5	2.09		24.31	97	1.79
**Sacubitril**	0.39	0.43	110.2	2.27		0.44	112.8	2.6
	3.12	3.35	107.3	3.61		3.31	106	2.56
	12.5	12.45	99.6	0.88		12.57	100.5	1.96
**LBQ657**	0.78	0.68	87.1	3.93		0.72	92.3	2.85
	6.2	6.23	100.4	2.27		5.96	95.3	3.5
	25	28.5	114	5.19		26.5	106	5.14
